# Rare haplotypes of the gene *TAS2R38* confer bitter taste sensitivity in humans

**DOI:** 10.1186/s40064-015-1277-z

**Published:** 2015-09-17

**Authors:** Emma E. Boxer, Nicole L. Garneau

**Affiliations:** Genetics of Taste Lab, Health Sciences Department, Denver Museum of Nature & Science, 2001 Colorado Blvd., Denver, CO 80205 USA; University of Colorado Denver, 1201 Larimer St., Denver, CO 80204 USA

**Keywords:** *TAS2R38*, Rare haplotype, PROP, Bitter, Taste, SNP

## Abstract

**Background:**

The *TAS2R38* gene is widely responsible for the well-known bimodal response to a family of bitter chemicals which includes 6-*n*-propylthiouracial (PROP). There are two common haplotypes of this gene, the recessive AVI and the dominant PAV, both of which are well studied. Conversely, the role of rare *TAS2R38* haplotypes on bitter taste sensitivity has been notoriously difficult to study due to small sample sizes. Here we present PROP sensitivity data of 97 individuals that have been observed to carry rare haplotypes (AAV, AAI, PAI, PVI) of the TAS2R38 gene.

**Results:**

Participants rated their bitter taste perception to a PROP filter disc then provided a buccal DNA sample from which the TAS2R38 gene was sequenced and analyzed. We found the prevalence of the PAV haplotype to be 42.3 %, AVI 53.1 %, AAV 2.5 %, AAI 1.2 %, PAI 0.8 % and PVI 0.1 %. We found that the AAI, AAV, and PAI haplotypes present intermediate taste sensitivity.

**Conclusions:**

These data are further evidence that bitter taste sensitivity to PROP exists as a broad range, and not exclusively as nontasters, medium tasters, and supertasters.

## Background

The ability of humans to detect bitter compounds begins with the 25 known genes that code for bitter receptors expressed by taste cells. These ligand-specific receptors constitute a family of proteins called T2R (Chandrashekar et al. 2000). Two ligands, PROP and phenylthiocarbamide (PTC), cause a well-studied bimodal response of a bitter sensation in some and “taste blindness” in others (e.g., Fox 1932).

This taste phenomenon was found to be primarily due to the *TAS2R38* gene. A 2003 report by Kim et al. shows that the two common haplotypes of the gene account for about 85 % of the bimodal taste response observed. Composed of 1002 nucleotides, *TAS2R38* contains three missense-coding single nucleotide polymorphisms (SNPs) at positions 145 (proline or alanine); 785 (alanine or valine), and 886 (valine or isoleucine) (Kim et al. 2003). Despite the eight possible amino acid combinations, only six haplotypes have been observed in the context of taste sensitivity; PAV and AVI are the common haplotypes, AAI and AAV are rare, PAI and PVI are extremely rare, and AVV and PVV have only been observed in disease-based population studies with no associated taste sensitivity data (Kim et al. 2003; Bufe et al. 2005; Mennella et al. 2011; Pemberton et al. 2009; Carrai et al. 2011). Typically, individuals with at least one copy of the dominant PAV haplotype have the ability to taste PTC/PROP, AVI homozygotes do not, and the rare haplotypes AAI and AAV have both been observed to confer an intermediate sensitivity (e.g., Kim et al. 2003; Bufe et al. 2005; Mennella et al. 2011, Garneau et al. 2014). Beyond these reports, taste sensitivity analysis of individuals with rare diplotypes has been scarce, with sample sizes too small to conduct statistics. Moreover, even when studies are large enough to capture rare haplotypes, they often use methodology that does not sequence all three SNPs, thus complicating the ability to interpret the haplotypes observed. To overcome this limitation in sequence analysis, we sequenced all three SNPs to confidently assess each haplotype. These data were then analyzed in the context of an individual’s PROP bitterness ratings. Here we report both the haplotype and diplotype frequencies observed, as well as the sensitivity of rare haplotypes relative to the common ones. As *TAS2R38* has been reported to contribute to nutritional choices (Duffy et al. 2010; Sandell et al. 2014; Hoppu et al. 2015), BMI (Tepper et al. 2014), cigarette and alcohol consumption (Keller et al. 2013; Hayes and Pickering 2012) and immunity (Lee et al. 2012), the data presented further advance the conversation on the role that rare haplotypes may be playing in not only taste, but in these important areas of human health.

## Methods

### Participants

Data from 1156 participants (age range of 18–93, 61 % female) were previously collected as part of a population study in the Genetics of Taste Lab at the Denver Museum of Nature & Science (Museum). Briefly, the citizen scientists in the original study facilitated data collection from a convenience sample of Museum guests, including buccal swabbing for DNA extraction and analysis, and standardized taste sensitivity ratings to a 0.453 M PROP filter disk (Garneau et al. 2014). The taste test was scored through trained use of a general Labeled Magnitude Scale (Green et al. 1996). The experiment took place in a sensory lab, and participants gave written informed consent as well as volunteered their time. All procedures were approved by the Western Institutional Review Board (Study No. 1109386, Protocol No. 2009 1028).

### *TAS2R38* SNP analysis and haplotype determination

Participant DNA was extracted from the buccal swabs. The 1002 nucleotide (nt) *TAS2R38* gene was amplified from nt 94 through nt 925 using PCR primers (Forward ACCAATGCCTTCGTTTTCTTGGTGA, Reverse TCACAGCTCTCCTCAACTTGGCA, Invitrogen). Sanger sequencing using the forward primer (High Throughput Genomics Center, Seattle, WA, USA, http://www.htseq.org) was conducted and the results were analyzed at the Museum using the software program Geneious. The forward read allowed for analysis of SNPs at positions 145 and 785. From this first assessment we could determine all samples that were AVI/AVI, PAV/AVI and PAV/PAV carriers. The remaining samples contained possible rare haplotypes. These were sequenced using both the forward and reverse primers, creating two reads that together fully captured all three SNPs. This analysis revealed 97 of our 1156 participants to be a carrier of a rare haplotype.

### Statistical analysis

Haplotype phase was resolved using the haplo.score package in R version 3.1.3. To normalize the taste sensitivity data, we transformed each raw PROP score using the log base 10. These normalized PROP score data were analyzed by one-way analysis of variance (ANOVA) and Tukey’s post hoc test to determine the differences in PROP ratings between all observed *TAS2R38* diplotypes. Analyses were performed using SYSTAT 13.

## Results

### Diplotype frequencies

The frequencies of each observed diplotype and haplotype are shown in Table 1.

**Table 1:** a *TAS2R38* haplotype, b diplotype and c frequencies and diplotype comparisons (ANOVA and Tukey’s Post Hoc)

	n	Frequency (%)
a Haplotype		
AVI	1227	53.1
PAV	978	42.3
AAV	59	2.5
AAI	28	1.2
PAI	18	0.8
PVI	2	0.1
b Diplotype		
PAV/AVI	461	39.9
AVI/AVI	356	30.8
PAV/PAV	242	20.9
AAV/PAV	27	2.3
AAV/AVI	24	2.1
AAI/PAV	5	0.4
AAI/AVI	15	1.3
PAI/AVI	15	1.3
AAV/AAV	3	0.3
AAI/AAV	2	0.2
AAI/AAI	2	0.2
AAI/PAI	2	0.2
PVI/PAI	1	0.1
PVI/PAV	1	0.1
Total	1156	100.0

Diplotype	AVI/AVI	PAV/AVI	PAV/PAV
c Significantly different (p < 0.05) than			
PAV/AVI	✓		
AVI/AVI		✓	
PAV/PAV	✓		
AAV/PAV	✓		
AAV/AVI	✓	✓	✓
AAI/PAV	✓		
AAI/AVI	✓		✓
PAI/AVI	✓		
AAV/AAV			✓
AAI/AAV			
AAI/AAI			
AAI/PAI			
PVI/PAI			
PVI/PAV			

### PROP sensitivity

**Fig. 1:**
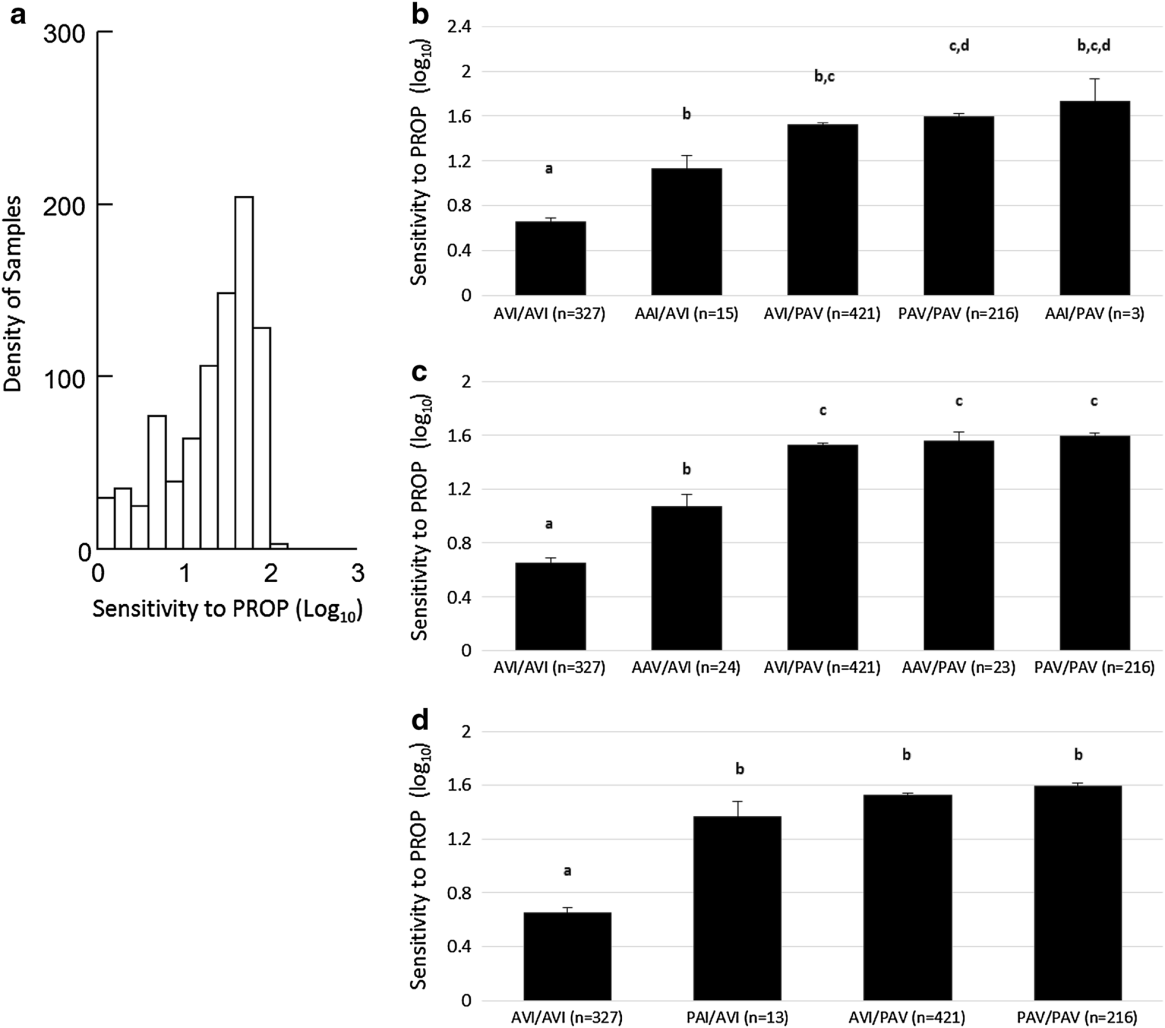
Comparison of taste sensitivity across *TAS2R38* diplotypes. *Shared letters* not significantly different (Tukey p < 0.05). Error bars reflect standard error. **a** Histogram showing range of log_10_ PROP scores, **b** AAI, **c** AAV and **d** PAI

The range of PROP scores (log_10_) of all subjects are shown in Fig. 1a. We hypothesized that rare haplotypes of the gene *TAS2R38* would fall within this wide range of sensitivity to the tastant PROP. Using ANOVA and Tukey’s post hoc test, we conducted genotype/phenotype relational analysis from participants with usable PROP data (n = 1051) to assess the role of three rare *TAS2R38* haplotypes, AAI (Fig. 1b) and AAV (Fig. 1c), and PAI (Fig. 1d) in bitter taste sensitivity. We included some additional sensitivity comparisons in Table 1c.

Here we show that AAI, AAV, and PAI confer taste sensitivity to PROP, supporting our hypothesis and confirming an earlier reported trend (Mennela et al. 2010). The following logic supports these conclusions. First, we found AAI/AVI has significantly greater sensitivity to PROP than the nontaster AVI/AVI (p = 0.02). It is clear that the AAI haplotype must confer this taste sensitivity in the AAI/AVI diplotype, since AVI does not. Second, AAI/AVI is significantly different from PAV/PAV (p = 0.03), but not AVI/PAV, suggesting that AAI has an intermediate taste sensitivity similar to that of AVI/PAV. Unsurprisingly due to the small sample size (n = 1), we could not infer any conclusions from AAI/AAI.

For the rare haplotype AAV, we found AAV/AVI to have a significantly greater sensitivity than AVI/AVI (p = 0.01), but lower sensitivity than AVI/PAV (p = 0.0) and PAV/PAV (p = 0.0), suggesting AAV confers an intermediate taste sensitivity as well, perhaps a weaker one than AAI since AAV/AVI is significantly less sensitive than AVI/PAV, but AAI/AVI is as sensitive as AVI/PAV. AAV/AAV (n = 3) had too small a sample size to infer any conclusions from.

For the haplotype PAI, we observed that PAI/AVI has a significantly greater sensitivity than AVI/AVI (p = 0.0), but no difference from AVI/PAV or PAV/PAV. This suggests that PAI confers a strong sensitivity, perhaps stronger than AAI and AAV, since these rare haplotypes did have significantly weaker sensitivity than PAV/PAV. Thus we propose a tentative relationship between these three haplotype sensitivities as AAV < AAI < PAI. The final rare haplotype, PVI, had sample sizes too small to analyze.

It should be noted here that although *TAS2R38* is known to be the primary influencer of PROP perception, there may be other cofactors not accounted for here, including other genes that have yet to be associated with taste, which may confound the data presented and should be examined in future studies.

In conclusion, we found AAI, AAV, and PAI to confer sensitivity to PROP, and these data provide needed evidence to fill the gap in knowledge on the role of rare haplotypes of the gene *TAS2R38* in bitter taste perception. Further, they substantiate a growing number of studies showing PROP sensitivity to be a normal range of responses from low to high sensitivity, and not a categorical grouping. Finally, this work contributes to the broader implications of *TAS2R38* variation in nutritional choices, lifestyle choices, and immunity.

## Abbreviations

PROP: 6-*n*-propylthiouracial
PTC: phenylthiocarbamide
SNP: single nucleotide polymorphism
Museum: Denver Museum of Nature & Science
nt: nucleotide
